# Cardiomyopathy care and real-world practice in the Netherlands

**DOI:** 10.1007/s12471-025-01956-1

**Published:** 2025-04-22

**Authors:** Pim van der Harst

**Affiliations:** Department of Cardiology, Division of Heart and Lungs, University Medical Centre Utrecht, Utrecht, The Netherlands

In this issue of the *Netherlands Heart Journal*, Groeneweg et al. present the Netherlands Society of Cardiology (NVVC) endorsement of the 2023 European Society of Cardiology (ESC) guidelines on the management of cardiomyopathies [1]. This document translates ESC recommendations into practical guidance for Dutch cardiologists, reflecting our national healthcare system, shared-care models, and expertise in cardiogenetics and imaging. The authors highlight key innovations, including the updated phenotypic classification and the shift from vague terms like ‘arrhythmogenic cardiomyopathy’, while emphasising multidisciplinary care. Notably, the NVVC endorsement provides practical modifications (e.g. tailoring the use of cardiac MRI and genetic testing) to support efficient, patient-centred application in Dutch practice.

This issue also features three original articles on current cardiology challenges. Bosman et al. [2] describe DZThuis, a pilot hospital-at-home model for acute decompensated heart failure. Comparable outcomes between home-based and in-hospital intravenous diuretics, suggest a promising, scalable approach amid rising demand for flexible care. Hopman et al. [3] explore the prognostic role of late gadolinium enhancement (LGE) on cardiac MRI in non-ischaemic cardiomyopathy patients with primary prevention ICD. They found no significant difference in ICD therapy between LGE+ and LGE− patients over five years, questioning LGE as a risk marker and supporting larger, multicentre studies to inform Dutch guidelines. Finally, van Bergeijk et al. [4] present long-term outcome data from the Netherlands Heart Registry (NHR), comparing surgical (SAVR) and transcatheter (TAVI) aortic valve interventions. Their study confirms ongoing differences in patient selection and outcomes: higher mortality with TAVI, largely due to comorbidity, and more frequent re-interventions after SAVR. These real-world data offer valuable insight into evolving treatment strategies.

This issue shows how evidence-based guidelines, local innovation, and national registry data can come together to support improved cardiovascular care.
